# Multifaceted Roles of Chemokine C-X-C Motif Ligand 7 in Inflammatory Diseases and Cancer

**DOI:** 10.3389/fphar.2022.914730

**Published:** 2022-06-28

**Authors:** Qianmiao Wu, Huaijun Tu, Jian Li

**Affiliations:** ^1^ Department of Hematology, Second Affiliated Hospital of Nanchang University, Nanchang, China; ^2^ Department of Medicine, Nanchang University, Nanchang, China; ^3^ Department of Neurology, Second Affiliated Hospital of Nanchang University, Nanchang, China

**Keywords:** CXCL7, CXCR1, CXCR2, inflammatory diseases, tumor

## Abstract

Over recent years, C-X-C motif ligand 7 (CXCL7) has received widespread attention as a chemokine involved in inflammatory responses. Abnormal production of the chemokine CXCL7 has been identified in different inflammatory diseases; nevertheless, the exact role of CXCL7 in the pathogenesis of inflammatory diseases is not fully understood. Persistent infection or chronic inflammation can induce tumorigenesis and progression. Previous studies have shown that the pro-inflammatory chemokine CXCL7 is also expressed by malignant tumor cells and that binding of CXCL7 to its cognate receptors C-X-C chemokine receptor 1 (CXCR1) and C-X-C chemokine receptor 2 (CXCR2) can influence tumor biological behavior (proliferation, invasion, metastasis, and tumor angiogenesis) in an autocrine and paracrine manner. CXCL7 and its receptor CXCR1/CXCR2, which are aberrantly expressed in tumors, may represent new targets for clinical tumor immunotherapy.

## 1 Introduction

In recent years, the role of chemokines in inflammatory diseases and tumors has received increasing attention (Van Damme et al., 2004). Chemokines are a family of small molecular weight proteins known for their ability to act as leukocyte chemotactic agents and are key regulators of the inflammatory response (Turner et al., 2007; White et al., 2013). For example, CXCL7 is a chemokine released by platelet activation, a potent chemotactic agent and neutrophil activator, with pro-inflammatory and pro-angiogenic effects (Walz et al., 1989). More recently, it has been found that CXCL7 is expressed not only by platelets, but also by neutrophils, megakaryocytes, natural killer cells, and lymphocytes, where it regulates their activation process and function, suggesting its important role in inflammation (Fu et al., 2004; Gleissner et al., 2008; Almeida et al., 2016). Furthermore, abnormal production of the CXCL7 chemokine has been identified in many inflammatory diseases, such as acute lung injury/acute respiratory distress syndrome, autoimmune diseases, graft-versus-host disease, and viral infections. However, the exact role of CXCL7 in the pathogenesis of inflammatory diseases is complex (Hughes-Austin et al., 2013; Bdeir et al., 2017; Yu et al., 2021).

Inflammation is an important component of the tumor microenvironment and a hallmark of cancer. Chemokines are key mediators in cancer-associated inflammation that can be present at sites of chronic inflammation, where they induce tumorigenesis or are secreted by tumor cells to form an inflammatory environment conducive to tumor development (Grivennikov et al., 2010; Greten and Grivennikov, 2019).

There is growing evidence indicating that CXCL7 is expressed by a variety of tumors, including renal cell carcinoma, breast cancer, and colorectal cancer, and that its aberrant expression can serve as a valid marker for multiple systemic malignancies, thus helping clinicians in early identification of malignancies and monitoring of treatment efficacy (Wang et al., 2013; Dufies et al., 2017; Li et al., 2021). By binding to CXCR1 and CXCR2, CXCL7 can affect tumor proliferation, invasion, migration, and tumor angiogenesis in an autocrine and paracrine manner through multiple signaling pathways (Wang et al., 2013; Mollica Poeta et al., 2019).

In this review, we summarize the role of CXCL7 in inflammatory diseases and tumors, describe the value of CXCL7 as a diagnostic and prognostic biomarker in inflammatory diseases and cancer, and discuss the importance of CXCL7 and its receptors as therapeutic targets.

## 2 The Biological Characteristics of CXCL7

CXCL7 is an ELR + CXC chemokine, a platelet-derived growth factor known as neutrophil-activating peptide 2 (NAP2). The basic proplatelet protein (PPBP) is the precursor protein of CXCL7, which is cleaved into the connective tissue activating peptide III (CTAP-III) and β-thromboglobulin antigen (βTG-Ag) (Walz et al., 1989; Malkowski et al., 1997; Schenk et al., 2002; Gleissner et al., 2008; Pilatova et al., 2013). CXCL7 exists as monomers, dimers and tetramers, which can be interconverted in different states (Brown et al., 2017a). Monomers predominate at lower concentrations while tetramers at higher concentrations. Dimers, which are the most preferred form in the glycosaminoglycan (GAG) bound state (Brown et al., 2017b). Recent studies have shown that CXCL7 also forms heterodimers with other platelet-derived chemokines and that GAG has a regulatory function in CXCL7 heterodimer and homodimer formation (Brown et al., 2017c).

The activity of CXCL7 is mediated by CXCR1 and CXCR2, members of the G protein-coupled receptor family with seven structural transmembrane domains (Gear and Camerini, 2003; Vandercappellen et al., 2008; Cheng et al., 2019). A main distinction between CXCR1 and CXCR2 is its selectivity for chemokines. CXCR1 binds to CXCL6 and CXCL8 with high affinity, but there is increasing evidence that CXCL7 is also the ligand for CXCR1 (Gear and Camerini, 2003). CXCR2 binds to CXCL1,CXCL2,CXCL3,CXCL5,CXCL6, CXCL7 and CXCL8 (Rajarathnam and Desai, 2020). CXCL7 has a stronger affinity for CXCR2 than CXCR1 (Ludwig et al., 1997). Tyrosine sulfation, a post-translational modification of chemokine receptors, regulates the receptor affinity for chemokine ligands (Moussouras et al., 2017). Different sites of tyrosine sulfation of CXCR1 and CXCR2 can result in changes in their respective affinities for CXCL7 (Moussouras et al., 2017). CXCR1 and CXCR2 are expressed in a variety of immune and non-immune cells, including tumor cells (Russo et al., 2014).

CXCL7 mainly has the following functions: i) promotes angiogenesis and acts as a potent chemotactic agent and activator of neutrophils; ii) regulates the expression of vascular endothelial growth factor (VEGF) C and D, two major growth factors of lymphatic vascular endothelial cells, and is involved in the lymphatic network. CXCL7 is also involved in cellular processes, such as DNA synthesis, glycolysis, mitosis, intracellular cAMP accumulation, prostaglandin E2 secretion, and hyaluronan and fibrinogen activator (Castor et al., 1983; Tai et al., 1992; Kalwitz et al., 2011).

### 3 The Relationship Between CXCL7 and Inflammatory Diseases

CXCL7 released by activated platelets induces the aggregation and activation of neutrophils and other immune cells, which play an important role in inflammatory diseases (Zarbock et al., 2007). In recent years, numerous studies have shown that CXCL7 is aberrantly expressed in various inflammatory diseases and has a strong correlation with pathogenesis (Hughes-Austin et al., 2013; Bdeir et al., 2017).

### 3.1 Acute Lung Injury/Acute Respiratory Distress Syndrome

Acute lung injury/acute respiratory distress syndrome (ALI/ARDS) is a severe unbalanced inflammatory response caused by multiple etiologies and respiratory disease with a high mortality rate (He et al., 2021). Its pathology is characterized by loss of alveolar epithelial and capillary barrier function, leading to alveolar infiltration of proteins and neutrophils, and a clinical presentation of intractable hypoxic respiratory failure (Amatullah et al., 2021; Quijada et al., 2021).

Previous studies have shown that CXCL7 is closely associated with the development and severity of ALI/ARDS disease. In a mouse model of ALI, deletion of CXCL7/PPBP strengthens the barrier function of the alveolar epithelium and attenuates neutrophil migration and activation, thus protecting mice from lung injury (Bdeir et al., 2017). In the bronchoalveolar lavage fluid (BALF) from patients with ALI, an increase in CXCL7/PPBP transcripts and proteins was accompanied by an increase in total protein and neutrophils, leading to a more severe state of ALI (Bein et al., 2021).

In recent years, a new coronavirus pneumonia (COVID-19) caused by SARS-CoV-2 has ravaged the world (Nazy et al., 2021). However, the clinical symptoms of SARS-CoV-2 infected patients are extremely variable, ranging from asymptomatic infection to acute pneumonia and even ARDS requiring mechanical ventilation support. The “cytokine storm” is characterized by an overproduction of cytokines and chemokines leading to an uncontrolled inflammatory response as a leading cause of ARDS. Chemokines CXCL10, IL6, CCL2, CXCL1, and CXCL5 have been shown to be up-regulated in patients with COVID-19, similar to that observed in the SARS and MERS epidemics (Coperchini et al., 2020). Patients who required admission to the intensive care unit had significantly higher circulating concentrations of CXCL10 and CCL2 than those with less severe clinical symptoms (Huang G. et al., 2020). Recently, studies have shown an increase in the expression of genes related to platelet activation in critically ill patients with COVID-19. The expression of CXCL7/PPBP was even more up-regulated in a disease severity-dependent manner (Yatim et al., 2021). The ROC curve analysis of CXCL7/PPBP showed that it was significant in predicting intubation ventilation treatment (area under the curve [AUC] = 0.8, *p* = 0.046), the optimal cut-off values for CXCL7 were 100% NPV and 45% PPV (Yatim et al., 2021). This suggests that CXCL7 may play a contributing role in the more critical forms of COVID-19.

Bronchopulmonary dysplasia (BPD) is a lung injury disorder that occurs after ventilator and oxygen therapy in preterm infants. Previous studies demonstrated the critical role of neutrophil- and pro-inflammatory cytokine-mediated inflammatory responses in the development of BPD (Choo-Wing et al., 2007; Fahy, 2009). Intratracheal transplantation of human umbilical cord blood (UCB)-derived mesenchymal stem cells (MSC) can attenuate hyperoxia-induced lung injury (Chang et al., 2011). Available studies have shown that hyperoxia significantly increases the expression of the inflammatory marker CXCL7 in lung tissue from neonatal rats. Early administration of MSCs for transplantation treatment significantly reduced CXCL7 expression (Chang et al., 2013). These findings suggest that CXCL7 may play a key role in the anti-inflammatory effects of MSC transplantation, and the main reason may be that CXCL7 mediates neutrophil aggregation in hyperoxic lung injury.

The presence of ALI/ARDS is a critical state of systemic inflammatory response syndrome (SIRS). The role of neutrophils in acute lung injury is well recognized, including cellular radicalization and release of reactive oxygen species (ROS) and protein hydrolysis granules (Moraes et al., 2006). NADPH oxidase 2 (NOX2) has an anti-inflammatory protective role in the pathogenesis of ARDS (Whitmore et al., 2013). In experimental models of SIRS in animals, only NOX2-deficient mice develop acute and severe lung lesions, including inflammation, diffuse thrombosis, and hemorrhage. Further investigation of the mechanism by which NOX2 deficiency induces ALI/ARDS showed that NOX2 deficiency can activate platelets to increase their release of CXCL7, induce neutrophil migration to the alveolar lumen, and the formation of extracellular neutrophil traps (NET), leading to ALI/ARDS (Hook et al., 2019). Consistent with these results, platelet activation-induced NET have been shown to cause acute lung injury associated with transfusions and influenza (Narasaraju et al., 2011; Caudrillier et al., 2012).

In the treatment of ARDS, the open lung approach (OLA), including the application of positive end-expiratory pressure (PEEP) and the lung recruitment maneuver (RM), has been shown to be beneficial in ARDS (Suzumura et al., 2014). OLA significantly improved pulmonary ventilation, arterial oxygenation, and gas exchange. Investigation of the mechanism of OLA revealed that OLA significantly reduced the level of platelet-derived chemokine CXCL7 in BALF, suggesting that OLA protects against lung injury by inhibiting platelet activation (Tojo et al., 2018). This may be related to platelet activation that improves pulmonary vascular thrombosis and CXCL7 release that leads to neutrophil infiltration into the alveolar lumen. This suggests that CXCL7 is a potential therapeutic target for lung injury.

### 3.2 Chronic Obstructive Pulmonary Disease

Chronic obstructive lung disease (COPD) is a chronic inflammatory disease of the airways and lung parenchyma associated with massive immune cell infiltration. Patients with severe COPD have increased expression of CXCL7 in the bronchial mucosa, as well as increased neutrophils and CXCR2 (Di Stefano et al., 2009). These neutrophils highly express CD44 and CD11b and can bind to intercellular adhesion molecule 1 (ICAM-1) and endothelial leukocyte adhesion molecule 1 (ELAM-1), which are highly expressed in the epithelium of the bronchial mucosa (Di Stefano et al., 1994). Another study showed an enhanced chemotactic response of monocytes to CXCL7 in COPD patients, mediated primarily by CXCR2, which can contribute to increased macrophage recruitment and activation in the lung (Traves et al., 2004). It is suggested that the CXCL7/CXCR2 axis induces the migration and adhesion of immune cells to the bronchial mucosa, leading to increased airway inflammation in COPD. Airway smooth muscle cells are a potent source of many cytokines and chemokine inflammatory mediators associated with local amplification of the airway inflammatory response. The inhibitor of I-kappa B kinase (IKK) in primary human airway smooth muscle (HASM) cells interfered with the NF-κB signaling pathway and thus significantly reduced CXCL7 transcription (Catley et al., 2006). Based on the important role of CXCL7 and HASM in the pathogenesis of COPD, IKK inhibitors and other NF-κB inhibitors can provide effective anti-inflammatory effects in patients with COPD.

### 3.3 Vascular Diseases

Alterations in chemokines can be detected during myocardial and cerebral ischemic infarction. On the one hand, chemokines recruit circulating leukocytes into the lesion and cause further damage, and on the other hand, they recruit stem cells to repair the damage (Gleissner et al., 2008).

CXCL7 induces neutrophil adhesion to blood vessels and transendothelial migration (Schenk et al., 2002). Leukocyte infiltration into the vessel wall is a key step in the development of hypertension. Alterations in CXCL7 can lead to a change in the inflammatory state in stress hypertension (Wu et al., 2020). Neutrophils play an important role in the development of delayed cerebral vasospasm (DCV) after aneurysmal subarachnoid hemorrhage. Neutrophils in cerebrospinal fluid (CSF) can act as predictors of DCV (Provencio et al., 2011). Anti-CXCR2 treatment significantly reduces infiltration of neutrophils into the infarcted area and decreases the infarct size (Oral et al., 2013).

Stem cell homing to the site of injury may play a reparative role. CXCL7 induces the homing of mesenchymal stem cells (MSCs) at the site of injury (Almeida et al., 2016). In chronic heart failure, reduced CXCL7 secretion from injured heart tissue impaired migration, survival and proliferation of cardiac stem cells (Boucek et al., 2015). This may be related to the regulation of angiogenesis by CXCL7 after binding to CXCR2 and stimulating the PI3K/AKT/mTOR pathway (Boucek et al., 2015). Circulating endothelial progenitor cells can be induced by the CXCL7/CXCR2 axis to the site of arterial injury and eventually differentiate into mature endothelial cells (Hristov et al., 2007a). Smooth muscle cells in the vasculature also promote endothelial regeneration after arterial injury through protein kinase C-delta (PKCδ)/signal transducer and activator of transcription 3 (STAT3)/CXCL7 paracrine mechanism (Ren et al., 2019). In stent interventions, these process may help promote stent endothelialization and prevent stenosis (Hristov et al., 2007b).

### 3.4 Inflammatory Bowel Disease

Inflammatory bowel disease (IBD), which includes Crohn’s disease (CD) and ulcerative colitis (UC), is associated with chronic inflammation of the gastrointestinal tract. In a rat model of UC, CXCL7 levels were significantly increased in intestinal tissues (Boshagh et al., 2019). Similarly in UC patients, CXCL7 levels were increased 13-fold (Kruidenier et al., 2006). CXCL7 is induced by matrix metalloproteinase 3 (MMP-3) and leads to subsequent neutrophil infiltration that promotes intestinal inflammation (Kruidenier et al., 2006). However, CXCL7 expression levels are low in CD, so monitoring intestinal tissue levels of CXCL7 may help distinguish between UC and CD (Kruidenier et al., 2006).

### 3.5 Rheumatoid Arthritis

Rheumatoid arthritis (RA) is a chronic autoimmune disease characterized by symmetric inflammatory polyarthritis (England et al., 2018). Several studies have shown that autoantibodies and other biomarkers, including chemokines and proteases are elevated in patients even years before the onset of RA (Hughes-Austin et al., 2013). CXCL7 was found to be significantly elevated in synovial fluid and serum of patients with RA 12 weeks before the onset of RA (Smolen et al., 2007). CXCL7, in combination with MMP3, can be used as a biomarker for RA (Guerrero et al., 2021). CXCL7 and CXCL4 are elevated in the synovium of early RA, which can help distinguish early RA from remitting arthritis (Yeo et al., 2016). The stimulator-of-interferon-gene (STING) signaling pathway limits Chikungunya virus (CHIKV) infection and postinfection arthritis, while STING deficiency leads to abnormal chemokine responses, including up-regulation of CXCL10 and down-regulation of CXCL5, CXCL7, and CXCR2, which promote the development of CHIKV arthritis (Geng et al., 2021).

Previous studies have shown that platelet-derived extracellular vesicles (PEVs) promote the migration and invasion of fibroblast-like synoviocytes (RA-FLS) in rheumatoid arthritis (Wang et al., 2021). CXCL7 is an important component of PEVs, and the use of the CXCR2 antagonist SB225002 partially prevents PEV-induced migration and invasion of RA-FLS. It can also reduce the phosphorylation of I-κB and NF-κB in RA-FLS (Wang et al., 2021), which in turn suggests that PEVs may activate the I-κB and NF-κB signaling pathway by binding CXCL7 to CXCR2, thus promoting migration and invasion of RA-FLS (Zhang et al., 2018; Wang et al., 2021).

### 3.6 Graft-Versus-Host Disease

Graft-versus-host disease (GVHD), both acute and chronic, remains a serious adverse immune reaction after allogeneic hematopoietic stem cell transplantation (HSCT), which poses a great life threat to transplantation (Zeiser and Blazar, 2017; Srinagesh and Ferrara, 2019; Hill et al., 2021).

CXCL7 has been shown to be involved in the immunomodulatory function of CD4^+^ T cells in acute GVHD (aGVHD) (Yu et al., 2021). The lower level of CXCL7 expression in CD4^+^ T cells of allogeneic HSCT mice compared to autologous HSCT was demonstrated by *in vitro* and *in vivo* experiments, whose results were consistent with those of RNA sequencing. Pathway enrichment analysis of CD4^+^ T cell differential genes in autologous and allogeneic HSCT revealed a non-negligible role of a chemokine signaling pathway in the signaling pathways related to immunomodulation related to aGVHD (Yu et al., 2021).

In a mouse model with chronic GVHD (cGVHD), four upregulated proteins were identified, including RAS and JUN kinase activators, CRKL, CXCL7, CCL8, and CCL9 chemokines (Du et al., 2018a). Although the application of low concentrations of CXCL7 antibodies for cGVHD showed a trend toward disease reduction, increasing the dose of CXCL7 antibodies did not further improve symptoms in cGVHD mice (Du et al., 2018a).

### 3.7 Inflammatory Demyelinating Diseases

Optic neuromyelitis optica (NMO) and multiple sclerosis (MS) are chronic and inflammatory idiopathic demyelinating diseases (IDDs). Until pathogenic anti-NMO antibodies were identified, NMO was believed to be a specific variant of MS (Lennon et al., 2004). Available studies have shown that CXCL1, CXCL5, and CXCL7 levels are significantly elevated in the CSF of NMO patients compared to MS patients and can be used as good bioindicators to differentiate NMO from MS, especially CXCL7 (Liu et al., 2020). Unlike MS, elevated neutrophil chemokines (CXCL7) and Th2 cell chemokines (CCL1, CCL22, and CCL17) were also found in the CSF of acute disseminated encephalomyelitis (ADEM), another inflammatory demyelinating disease, helping to differentiate ADEM from MS (Franciotta et al., 2006).

### 3.8 Other Inflammatory Diseases

CXCL7 levels, which are expressed at elevated levels in the CSF of patients with neurosyphilis, may participate in the pathogenesis of neurosyphilis by altering the permeability of the blood-brain barrier and promoting the spread of *T pallidum* (Li et al., 2020). However, CXCL7 has only moderate diagnostic efficiency for neurosyphilis (Li et al., 2020). CXCL7 concentration was also correlated with total protein levels in neurosyphilis CSF (Li et al., 2020). Urokinase plasminogen activator (uPA) concentrations were significantly higher in neurosyphilis CSF than in non-neurosyphilis. Furthermore, uPA levels were found to be correlated with total protein levels (Lu et al., 2016). CXCL7 is a strong neutrophil activator, and activated neutrophils release uPA (Abraham et al., 2003), suggesting that CXCL7 may participate in neurosyphilis by inducing and activating neutrophils to release uPA, thus increasing the protein content in the CSF of neurosyphilis patients.

In allergic pulmonary inflammation, leukotrienes D4 (LTD_4_) inhibit platelet activation and CXCL7 release by blocking the leukotrienes C4 (LTC_4_) activated cysteinyl leukotriene receptor 2 (CysLT_2_R) signaling pathway, thus limiting the severity of allergic pulmonary inflammation (Liu et al., 2021). Further studies have shown that LTD_4_ also blocks LTC_4_-induced phosphorylation of three kinase families, extracellular signal-regulated kinase (ERK), c-Jun N-terminal kinase (JNK), and mitogen-activated protein kinase (p38 MAPK), of the mitogen-activated protein kinase (MAPK) signaling pathway in platelets (Liu et al., 2021). Furthermore, a selective inhibitor of p38 (SB203580) blocks LTC_4_-induced CXCL7 release, suggesting that it may be regulated by the p38 MAPK signaling pathway.

In patients with autoimmune thyroid disease, monocytes are in an abnormal pro-inflammatory state, as evidenced by the overexpression of Ly6C^low^ clone monocytes (Nikolic et al., 2005). Previous studies have shown that significant upregulation of CXCL7/PPBP expression was associated with a pro-inflammatory state of monocytes (van der Heul-Nieuwenhuijsen et al., 2010). Antiphospholipid syndrome (APS) is an autoimmune thrombotic disorder, has been associated with a significantly higher plasma levels of platelet activation-related chemokines CXCL4, CXCL7, and CCL5, suggesting a role for CXCL7 in APS thrombotic events (Patsouras et al., 2015). Higher plasma levels of CXCL7 have also been found in other autoimmune diseases, such as Sjogren syndrome, Vogt-Koyanagi-Harada syndrome (VKH), and vitiligo (Egerer et al., 2006; Liang et al., 2019; Egbeto et al., 2020), further indicating that CXCL7 could play an important role in the pathogenesis of autoimmune diseases that should be further studied in more detail.

### 4 The Relationship Between CXCL7 and Neoplastic Diseases

Inflammation is an important component of the tumor microenvironment; The pro-inflammatory activity of CXCL7 is also manifested in a variety of tumorigenic diseases (Chatterjee et al., 2015). Moreover, CXCL7 can regulate tumor cell proliferation, invasion, and migration through multiple pathways (Wang et al., 2013; Mollica Poeta et al., 2019). The proangiogenic function of CXCL7 also provides favorable conditions for tumor cell survival. The expression, specific functions, and therapeutic targeting of CXCL7 in different types of cancers are discussed in detail in the following sections (Table 1).

**TABLE 1:** Dysregulation of CXCL7 expression and its functions in the tumor microenvironment.

Tumor type	Expression of CXCL7	Effects of CXCL7 on tumor biological properties	Effects of CXCL7 on immune cell infiltration in tumor microenvironment	References
Pancreatic cancer	↓	NA	NA	Matsubara et al. (2011)
Colorectal cancer	↑	Promotes tumor metastasis and angiogenesis	NA	Li et al. (2019), Li et al. (2021), Desurmont et al. (2015)
Cervical cancer	↓	NA	NA	Grisaru et al. (2000), Xie et al. (2015)
Ovarian cancer	↓	NA	Recruitment of CD8^+^ T cells	Clarke et al. (2011), Karapetsas et al. (2018)
Myelodysplastic syndrome	↓	NA	NA	Aivado et al. (2007)
Acute lymphoblastic leukemia	↓	NA	NA	Shi et al. (2009)
Lung cancer	↑	NA	Recruitment of macrophages and helper T cells	Yee et al. (2009), Lee et al. (2011), Ulivi et al. (2013), Unver (2021), Unver et al. (2015), Huang et al. (2016),Ye et al. (2016)
Renal cell carcinoma	↑	Promotes tumor cell proliferation, stemness and angiogenesis	NA	Dufies et al. (2017), Kinouchi et al. (2017), Grépin et al. (2014), Strieter et al. (2006), Huang et al. (2018)
Cholangiocarcinoma	↑	Promotes tumor cell proliferation, invasion and metastasis	NA	Guo et al. (2017), Sueoka et al. (2014)
Breast cancer	↑	Promotes tumor cell proliferation, invasion, metastasis and stemness	Recruitment of neutrophils	Wang et al. (2013), Yu et al. (2010), Yu et al. (2007)
Gliomas	↑	Promotes tumor cell metastasis and stemness	Recruitment of macrophages	Yan et al. (2021), Bournazos et al. (2016), Sironi et al. (2006)
Gastric Cancer	↑	Promotes tumor cell invasion and metastasis	Recruitment of helper T cells	Yamamoto et al. (2019), Kinoshita et al. (2015)
Bladder cancer	↑	NA	Recruitment of dendritic cells, neutrophils, and CD8^+^ T cells	Sun et al. (2021)

↓, Down-regulation; ↑, Up-regulation; NA, no published data available.

### 4.1 Abnormal Expression of CXCL7 in Tumors

CXCL7 is aberrantly expressed in a variety of tumors of the digestive system. Plasma levels of CXCL7 are significantly reduced in patients with pancreatic cancer, an alteration that occurs at any stage of pancreatic cancer, including the early stages (stages I and II) (Matsubara et al., 2011). CXCL7 is a new biomarker independent of CA19-9 (Matsubara et al., 2011). In contrast, in patients with CRC, the plasma levels of CXCL7 was found to be significantly higher compared to healthy controls (Li et al., 2019). The ROC curve was used to evaluate the clinical diagnostic efficacy of CXCL7 and other CRC tumor markers CEA, CA19-9, and CA125, and the results showed that CXCL7 had the highest diagnostic power, with an AUC of 0.862 (95% CI: 0.831–0.890) (Li et al., 2019), thus indicating that abnormal levels of CXCL7 may contribute to early detection and diagnosis of intestinal system tumors.

CXCL7 may also play an important role in the early diagnosis of gynecological tumors. CTAPIII/CXCL7 is expressed in normal cervical epithelial cells; however, its expression gradually disappears with the appearance and progression of cervical cancer (Grisaru et al., 2000). Another study reported that in early ovarian cancer, the expression of CTAPIII/CXCL7 decreased (Clarke et al., 2011). CXCL7 not only significantly improves the diagnostic sensitivity of CA125 to early ovarian cancer, but also helps with the diagnosis of ovarian cancer that does not express CA125 (Clarke et al., 2011).

For hematological diseases, a decrease in plasma CXCL7 levels can be used as a diagnostic marker for advanced disease of myelodysplastic syndrome refractory anemia with excess blasts (RAEB) and RAEB in transformation (RAEB-t) (FAB classification) or RAEB-1 and RAEB-2 (WHO classification) (Aivado et al., 2007). Decreased CXCL7 levels can also be used to differentiate pediatric acute lymphoblastic leukemia (ALL) patients from healthy controls and pediatric acute myeloid leukemia (AML) patients (Shi et al., 2009). Bone marrow stromal cells can induce monocytes to secrete CXCL7, which in turn regulates CXCL7 expression in megakaryocytes (Pillai et al., 2006). CXCL4 and CXCL7 promote the survival of hematopoietic progenitor cells and reduce their chemosensitivity to cytotoxic drugs (Han et al., 1997). Excess CXCL4 and CXCL7 in the bone marrow microenvironment contribute to the resistance towards decitabine in chronic monocytic leukemia (Meldi et al., 2015).

In the respiratory system, CXCL7 has been found to be significantly elevated 29 months prior to lung cancer development, making it the first blood biomarker capable of diagnosing stage 0 cancer (Yee et al., 2009; Lee et al., 2011; Ulivi et al., 2013). The concentration of CXCL7 is significantly higher in the blood draining tumors from patients with lung cancer compared to peripheral blood, and the CXCL7 gradient was correlated with the absolute number of helper T cells (r = 0.49, *p* = 0.03) (Yee et al., 2009; Spaks, 2017). CTAPIII/CXCL7 levels decreased after radical surgery, which in turn suggested that CXCL7 is useful for postoperative monitoring of small residual lesions and the prediction of disease recurrence (Yee et al., 2009). When comparing bronchial airway epithelial cell samples from mice with early stage lung squamous cell carcinoma (LSCC) with those of normal mice, CXCL7 gene is overexpressed 115-fold, indicating that CXCL7 is not only a blood biomarker for the diagnosis of non-small cell lung cancer (NSCLC), but also a biomarker for bronchial brush samples (Xiong et al., 2017). Furthermore, Du et al. (2018b) found that CXCL7 had a higher diagnostic efficacy for NSCLC (AUC of ROC: 0.806, 95% CI: 0.748–0.863) compared to traditional diagnostic biomarkers for lung cancer (CEA, SCCAg, and Cyfra211) (Du et al., 2018b). These studies have consistently suggested that CXCL7 can be used as a diagnostic marker for early-stage lung cancer (especially stage 0 lung cancer).

In the urinary system, Kinouchi et al. (2017) demonstrated that the expression of the CXCL7 gene in the peripheral blood from patients with renal cell carcinoma (RCC) was significantly higher than that of healthy controls and could be used as an independent diagnostic marker for RCC. In particular, CXCL7 was elevated from stage pT1a in patients with RCC and gradually increased with the stage TNM of RCC.

### 4.2 Effects and Molecular Mechanisms of CXCL7 on Tumor Biological Behavior

#### 4.2.1 Renal Cancer

In clear cell renal cell carcinoma (ccRCC), mutations in the von Hippel-Lindau gene lead to overexpression of VEGF, making ccRCC a highly vascularized tumor (Hutson et al., 2010; Wallace et al., 2016). The pro-inflammatory pro-angiotropic factor CXCL7 was shown to promote ccRCC cell proliferation *in vitro* and tumor growth *in vivo* (in nude mice) (Grépin et al., 2014). In contrast, an inhibitor of the CXCL7 receptors CXCR1 and CXCR2 (SB225002) inhibited the proliferation of ccRCC cells and endothelial cells, thereby strongly suppressing tumor growth and aberrant tumor angiogenesis (Grépin et al., 2014). The central pro-inflammatory cytokine interleukin 1β (IL-1β) induces increased CXCL7 secretion by ccRCC cells, which can directly act on the surface receptor CXCR1/2 of ccRCC cells (Grépin et al., 2014); CXCL7 also acts paracrinely by promoting the proliferation of RCC cells, which in turn promotes further secretion of CXCL7 by peripheral blood monocytes in ccRCC (Kinouchi et al., 2017).

**FIGURE 1:**
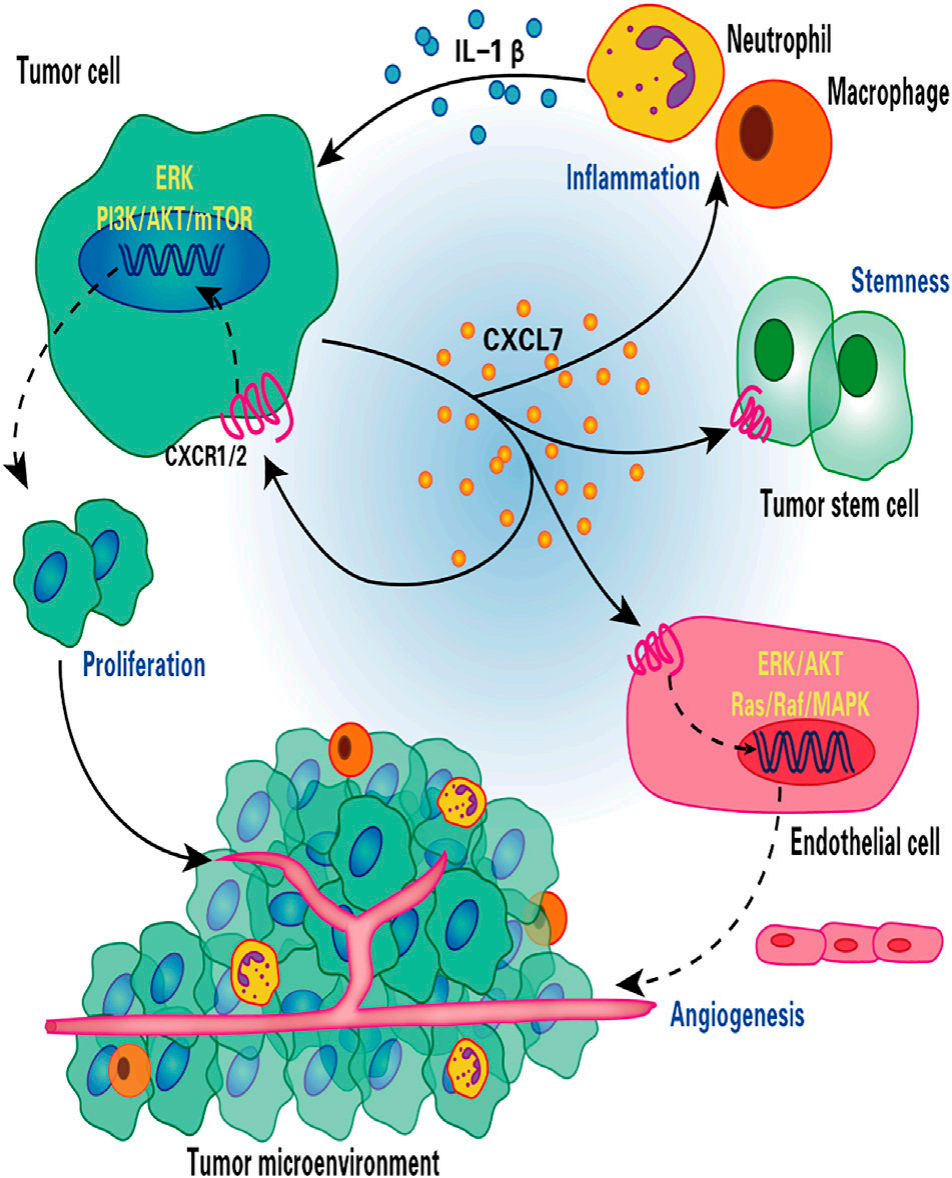
Role of CXCL7 in renal cell carcinoma. CXCL7 secreted by renal cancer cells binds to CXCR1/2 on the surface of tumor cells and endothelial cells, stimulating tumor cell and endothelial cell proliferation and tumor angiogenesis through ERK/AKT and Ras/Raf/MAPK signaling cascades, respectively. CXCL7 also maintains tumor stem cell properties and recruits neutrophils and macrophages to infiltrate, while inflammatory cells secrete IL-1β in turn promotes CXCL7 secretion by renal cancer cells, forming a positive feedback loop. Thus, the CXCL7/CXCR1/CXCR2 signaling axis is critical for tumor growth and angiogenesis and for maintaining the tumor inflammatory environment.

Mechanistically, CXCL7 enhances the activity of the PI3K/AKT/mTOR and ERK cell proliferation signaling pathways by binding to the CXCR1 and CXCR2 ccRCC cell membrane receptors and exerting a proliferative effect on tumor cells (Figure 1) (Grépin et al., 2014). CXCL7 also acts through the Ras/Ras/MAPK signaling pathway associated with tumor angiogenesis (Figure 1) (Strieter et al., 2006). Treatment of nude mice bearing tumors using anti-CXCL7 antibodies may inhibit tumor growth in three ways (Grépin et al., 2014): i) it decreases the activity of ERK and AKT signaling pathways; ii) it decreases the number of Ki-67-labeled tumor cells and increases the number of necrotic regions; iii) it decreases carbonic anhydrase 9 (CA Ⅸ) expression and the hypoxic regions associated with the induction of invasive cell production, which results in the inhibition of tumor growth of ccRCC.

According to previous studies, tumor stem cells have an extreme capacity for self-renewal and tumorsphere formation, promoting tumor formation and metastasis, and participating in the development of tumor drug resistance (Lytle et al., 2018; Huang T. et al., 2020). Galectin-3 (Gals-3) maintains the properties of RCC tumor stem cells. Studies on the mechanism of action of Gals-3 revealed that down-regulation of Gals-3 inhibits the expression of CXCL7 and CXCR2, thus suppressing tumor formation and colony formation capacity of renal cancer cells. Conversely, up-regulation of CXCR2 expression restores the tumorigenic properties of Gals-3 knockdown cells (Huang et al., 2018). These results suggest that the CXCL7/CXCR1/CXCR2 axis may represent an effective target for the treatment of RCC. In addition, Dufies et al. (2017) have identified CXCL7 as a valid predictor of the efficacy of sunitinib in clear cell RCC.

The CXCL7/CXCR2 axis promotes early metanephric development. Thus, active CXCL7/CXCR2 signaling maintains nephroblastoma survival (Levashova et al., 2007). CXCL7 induces tumor angiogenesis and invasiveness through upregulation of matrix metalloproteinase 9 (MMP9) and endothelial and thylakoid markers (Levashova et al., 2007). Nephroblastoma is sensitive to CXCR2 antagonist-induced apoptosis (Levashova et al., 2007). The CXCL7/CXCR2 axis could be a new target for nephroblastoma therapy.

#### 4.2.2 Cholangiocarcinoma

The expression of CXCL7 is higher in tissues with cholangiocarcinoma (CC) than in adjacent nontumor tissues, and the high expression of CXCL7 is significantly associated with poor differentiation, lymph node metastases, vascular invasion, and advanced clinical stages of CC. Furthermore, the overall survival (OS) of patients with CC with high expression of CXCL7 was significantly shorter (Guo et al., 2017). *In vitro* studies showed that CXCL7 and CXCR2 were highly expressed in CC cell lines HuCCT1, HuH28, QBC939, EGI-1, OZ and WITT, and overexpression or knockdown of CXCL7 promoted or inhibited proliferation and invasion of CC cells, respectively, suggesting that CXCL7 can act in an autocrine mode in tumors (Guo et al., 2017). Furthermore, the addition of exogenous CXCL7 medium or conditioned medium overexpressing CXCL7 human liver stellate cells also promoted invasiveness of QBC939 cells, indicating that CXCL7 could also act paracrinely in CC (Guo et al., 2017). Sueoka et al. (Sueoka et al., 2014) also demonstrated that in the intrahepatic cholangiocarcinoma (ICC) cell lines RBE and SSP25, both CXCR2 siRNA and SB225002 significantly inhibited cell proliferation, migration, and invasion, while SB225002 also inhibited the growth of subcutaneous transplanted tumors in nude mice.

The AKT signaling pathway has been shown to be involved in human tumorigenesis and development, while the overexpression of CXCL7 in cholangiocarcinoma increased the activity of the AKT signaling pathway, suggesting that the AKT signaling pathway is involved in the malignant phenotype mediated by the CXCL7/CXCR2 axis of cholangiocarcinoma cells (Guo et al., 2017; Cheng et al., 2019). At the site of metastasis, a microenvironment conducive to disease progression is formed by the interaction of tumor cells and host cells, called a metastatic niche. Leukocytes have been shown to be recruited to the metastatic niche and support metastasis (Qian et al., 2011; Spicer et al., 2012). Tumor cells can adhere to and activate platelets and recruit neutrophils through the CXCL5/CXCL7/CXCR2 axis to promote the formation of early metastatic niches (Labelle et al., 2014).

#### 4.2.3 Breast Cancer

**FIGURE 2:**
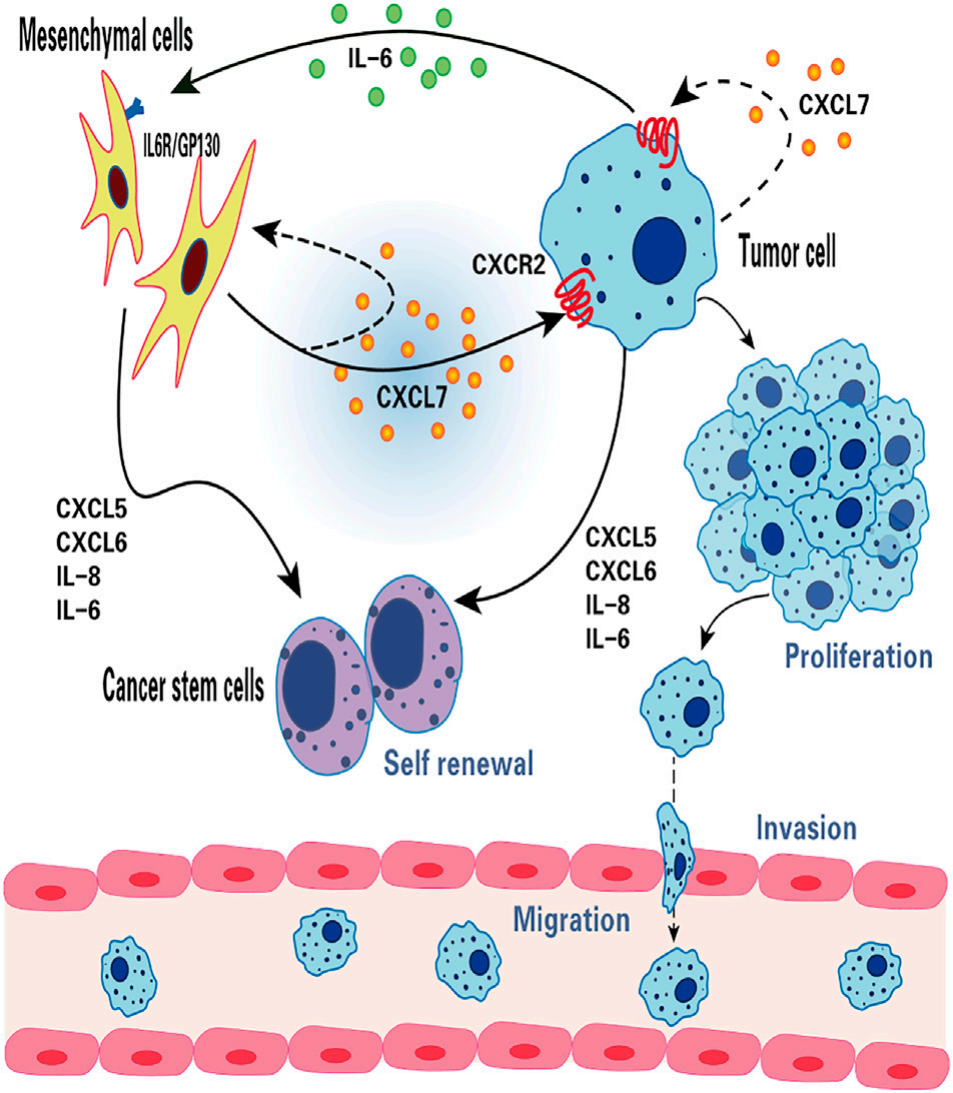
The function of CXCL7 in breast cancer. CXCL7 mediates the interaction between MSCs, breast cancer cells and breast cancer stem cells through IL-6 and IL-8, CXCL6 and CXCL5 cytokine networks. CXCL7 promotes tumor cell proliferation, migration and invasion by autocrine or paracrine means, attracts MSCs into the tumor niche and maintains the self-renewal capacity of tumor stem cells.

In primary breast cancer, CXCL7 concentration was found to be significantly correlated with Ki67 expression, indicating that CXCL7 was associated with the active proliferation of tumor cells (Wang et al., 2013). There is growing evidence that MSCs and cytokines in the tumor microenvironment (TME) can influence tumorigenesis and progression (Whiteside, 2018; Yin et al., 2020; Choi et al., 2021). Previous studies using *in vitro* and mouse xenograft models revealed that bone marrow MSCs can accelerate tumor growth by increasing the self-renewal capacity (number) of breast cancer stem cells (CSCs), and this effect depends on the regulation of the intercellular IL-6/CXCL7 cytokine network (Liu et al., 2011). Among them, IL six produced by breast cancer cells acts on IL-6R/GP130 receptors in bone marrow MSCs to stimulate the production of CXCL7 by bone marrow MSCs. In turn, CXCL7 induces breast cancer cells and MSCs to secrete a large number of cytokines, including IL-6, IL-8, CXCL6, and CXCL5, which then induce an increase in the population of breast CSCs. In addition, increased IL-6 interacts with MSCs to form a positive feedback loop (Liu et al., 2011). These results suggest that the IL-6/CXCL7 network in the TME plays an important role in the growth of breast cancer (Figure 2).

CXCL7 promotes the growth, invasion, and metastasis of breast cancer. Malignant breast cancer cells (MCF10CA1a.cl1) expression of both CXCL7 and CXCR2 is higher than that of precancerous cells (MCF10AT), where MCF10AT transfected with CXCL7 were significantly more invasive through the basement membrane of the stroma, an effect that was markedly inhibited by the use of the CXCL7 antibody (Tang et al., 2008). CXCL7 is confirmed to play a role in breast cancer invasion (Figure 2).

Zcharia et al. (2001) found that heparanase activity was closely related to the invasive and metastatic ability of breast cancer cells. Heparanase activity was significantly higher in malignant breast cancer cells than in MCF10AT cells and increased in MCF10AT cells after transfection with CXCL7 (Tang et al., 2008). In addition, Yu et al. (2010) found that CXCL7 siRNA inhibited the heparanase activity and invasive ability of CXCL7-transfected MCF10AT cells. In breast cancer, CXCL7 also induced the expression of lymphangiogenic factors VEGF-C and VEGF-D and promoted the lymphatic spread of tumor cells. The ability to secrete VEGF-C and VEGF-D and the invasive capacity of MCF10AT transfected with CXCL7 were also decreased by acting with the selective CXCR2 inhibitor SB225002 (Yu et al., 2007; Yu et al., 2010). These results suggest that CXCL7 facilitates breast cancer invasion and metastasis by increasing heparanase activity and promoting the secretion of VEGF-C and VEGF-D in breast cancer cells.

Low concentrations of piperine fractional piper nigrum extract (PFPE) have also been shown to exert anticancer immune effects in breast cancer rats. PEPE promotes Th1 expression, inhibits Th2, Treg, and CXCL7 expression, and reduces neutrophil infiltration, thus significantly reducing breast cancer tumor volume without side effects on blood biochemical parameters in rats (Saetang et al., 2021). Interestingly, the components of PFPE, 3-carene and pellitorine cause tumor regression by inhibiting CXCL7 production mediated by the activator IL-6 (Basholli-Salihu et al., 2017). It is shown to mediate antitumor effects through the regulation of CXCL7.

#### 4.2.4 Colorectal Cancer

In CRC, high expression of CXCL7 is closely associated with tumor vascular production. Previous studies have shown that CXCL7 is positively correlated with VEGF and that the coexpression of CXCL7 and VEGF is a risk factor for a poor prognosis in CRC patients (Li et al., 2021).

Patients with obstructive CRC (OCRC) have a higher risk of surgery requirement and a worse prognosis than patients with non-obstructive CRC (non-OCRC). According to previous studies, serum CXCL7 concentrations are not only higher in patients with OCRC compared to healthy controls, but also in patients without OCRC (Li et al., 2019). CXCL7 was reported to have the highest diagnostic efficiency (AUC of 0.918, sensitivity of 86.54% and specificity of 81.87%) compared to existing diagnostic indicators for OCRC (CEA, CA199, CA125) (Li et al., 2019). Serum CXCL7 levels in OCRC patients have also been associated with stage N lymph node metastasis (N0-N2) and TNM stage (I-II to IV), and analysis of the multifactorial Cox proportional risk regression model analysis showed that high serum CXCL7 levels were an independent factor for poorer OS in OCRC patients (HR = 2.216, *p* = 0.032) (Li et al., 2019). These results suggest that serum CXCL7 is a potential biomarker for the diagnosis and prognosis of patients with OCRC, which can help improve the early diagnosis of OCRC, reduce the risk of acute surgery, and improve patient survival.

The expression of CXCL7 and CXCR2 was found to be negatively correlated with disease-free survival (DFS) and OS in patients with liver metastatic CRC, suggesting that the CXCL7/CXCR2 signaling pathway may be involved in tumor recurrence and progression (Desurmont et al., 2015). 5-fluorouracil-based neoadjuvant chemotherapy regimens increased CXCL7 and CXCR2 gene expression in patients with CRC developing liver metastases, thus suggesting that neoadjuvant chemotherapy may have a role in promoting the spread of CRC cells *in vivo* through the CXCL7/CXCR2 axis (Desurmont et al., 2015).

#### 4.2.5 Gliomas

Increased infiltration of myeloid cells, particularly myeloid-derived suppressor cells (MDSCs) and tumor-associated macrophages (TAMs), in the TME is associated with glioma progression and resistance to anti-angiogenic therapies (AATs) (Hussain et al., 2006; Sica et al., 2006; Lu-Emerson et al., 2013). Investigations have shown that CSF1-CSF1R signaling is associated with monocyte proliferation and differentiation and plays an important role in tumor angiogenesis and progression (Priceman et al., 2010; Hume and MacDonald, 2012; Zhu et al., 2014). The use of CSF1R inhibitors (GW2580) reduces myeloid cells in the TME of gliomas and significantly decreases the expression of chemokine CXCL7, thus inhibiting tumor growth (Achyut et al., 2015). The ERK-MAPK pathway plays a regulatory role in the expression of CXCL7 by myeloid cells and is correlated with the Ki67 proliferation data (Achyut et al., 2015).

In gliomas, Fibrinogen-like protein 2 (FGL2) secreted by glioma cells recruits macrophages to the TME and induces CXCL7 (Yan et al., 2021). *In vitro*, CXCL7 improves tumor stem cell properties, including tumorsphere formation capacity and migration ability; *in vivo*, CXCL7 increases the incidence and shortens the survival time of gliomas in mice (Yan et al., 2021).

Additional studies have shown that FGL2 secreted by glioma cells activates the downstream signaling pathway Syk/PI3K/AKT/HIF1 by binding to CD16a receptors on the surface of macrophages, thus mediating the release of CXCL7 from macrophages and further promoting the development and progression of glioma of glioma by CXCL7 (Sironi et al., 2006; Bournazos et al., 2016; Yan et al., 2021). Glucocorticoids and macrophage-inactivating factors IL-4 and IL-10 were also found to significantly downregulate CXCL7 mRNA and protein levels in mononuclear phagocytes (El-Gedaily et al., 2004).

#### 4.2.6 Other Neoplastic Diseases

Infiltration of various immune cells has been shown to be strongly associated with the biological behavior of tumors and the clinical outcome of patients with cancer (Sarode et al., 2020; Sui et al., 2020). Furthermore, the recruitment of immune cells is largely dependent on the expression of chemokines (Li et al., 2018; Strazza and Mor, 2020). The infiltration of CD8^+^ T lymphocytes has been shown to be an independent and good prognostic factor in epithelial ovarian cancer (Sato et al., 2005). The transcription factor myeloid ecotropic viral integration site 1 (MEIS1) binds to a specific region on the promoter of the CXCL7 gene and induces the synthesis and secretion of CXCL7 in cells of ovarian cancer, thus triggering the recruitment of CD8^+^ T lymphocytes in ovarian cancer (Karapetsas et al., 2018). Yamamoto et al. (2019) found that CXCL7 was expressed in 36.4% of 590 patients with gastric cancer and was correlated with clinicopathological characteristics such as diffuse gastric cancer, lymph node metastasis, lymphovascular, and vascular invasion. The high expression of CXCR1 and the low expression of CXCR2 in gastric cancer were shown to be associated with higher levels of AFP, greater tumor size, and a higher stage of TNM tumor stage (Chen et al., 2020). After surgery in patients with gastric cancer, CXCL7 levels in peripheral blood and tumor drainage blood were higher in the recurrence group than in the group without recurrence, and the concentration gradient of CXCL7 was correlated with the absolute number of helper T cells in TMN (Chen et al., 2020). Inhibition of CXCL7 expression in gastric cancer cells by siRNA significantly reduced their migration ability (Chen et al., 2020). Significantly elevated levels of CXCL7 were obtained in tissues of bladder cancer (BLCA) by bioinformatics analysis (Sun et al., 2021). CXCL7 may regulate protein kinase N1 and G protein-coupled receptor kinase 2, and the mRNA encoding CXCL7 is targeted and regulated by miR-154, miR-487, miR-525, and miR-524 (Sun et al., 2021). According to previous studies, the expression of CXCL7 was associated with infiltration of dendritic cells, neutrophils and CD8 + T cells in BLCA (Sun et al., 2021). CXCL1, CXCL5, CXCL7, and CXCL8 expression in NSCLC have been associated with low patient survival, and CXCL7 is positively correlated with macrophage infiltration in TME, while its receptor CXCR2 is positively correlated with both neutrophil and macrophage infiltration (Unver, 2021). It is well known that M2-type TAMs are often associated with tumor progression. M2-type TAMs increase the proliferation and migratory capacity of Lewis lung cancer (LLC) cells (Lamagna et al., 2006; Zhang et al., 2012). In lung adenocarcinoma, M2-type macrophages are also associated with accelerated lymphangiogenesis (Zhang et al., 2012). In the LLC model, overexpression of CXCL7 chemokine induced increased M2-polarized macrophage infiltration and accelerated early LLC tumor development (Unver et al., 2015). This suggests that CXCL7 chemokines can influence immune cell infiltration and inflammatory responses in the TME, which in turn affects tumor progression as well as clinical treatment and outcomes of tumor patients.

Tumor cells are often present in the hypoxic TME, and hypoxia can induce changes in chemokines in the TME, which affects the biological behavior of the tumor (Korbecki et al., 2021). Chronic hypoxia increases the expression of CXCL7 in cervical cancer cells and astrocytes (Xie et al., 2015; Samy et al., 2018). However, chronic hypoxia does not influence CXCL7 expression in lung adenocarcinoma cells and HepG2 hepatocellular carcinoma cells (Huang et al., 2016; Ye et al., 2016). CXCR1 and CXCR2 act as receptors for CXCL7, and hypoxia can affect the function of CXCL7 by affecting the expression of the CXCL7 receptor. Chronic hypoxia increases the expression of CXCR1 and CXCR2 in prostate cancer cells and cervical cancer cells, and this effect is based on hypoxia-inducible factors 1 (HIF-1) and NF-κB activation (Maxwell et al., 2007; Liu et al., 2014; Taniguchi and Karin, 2018). Chronic hypoxia also increased CXCR2 expression in aortic endothelial cells, suggesting that hypoxia influences tumor angiogenesis by increasing the sensitivity of endothelial cells to CXCR2 ligands (Moldobaeva and Wagner, 2005). However, this effect varies depending on the type of tumor, e.g., chronic hypoxia decreased CXCR2 expression in gastric cancer cells (Kinoshita et al., 2015). Hypoxia and hypoxia-activated signaling pathways are effective targets in cancer therapy, where the former can cause alterations in the expression of CXCL7 and its receptor CXCR1/2 in tumors. Consequently, targeting CXCL7 and its receptor has the potential to be used as a new oncology therapy (Jing et al., 2019; Ivan et al., 2021).

## 5 Intracellular Signaling Pathways of CXCL7

### 5.1 The PI3K/AKT/mTOR Signaling Pathway

**FIGURE 3:**
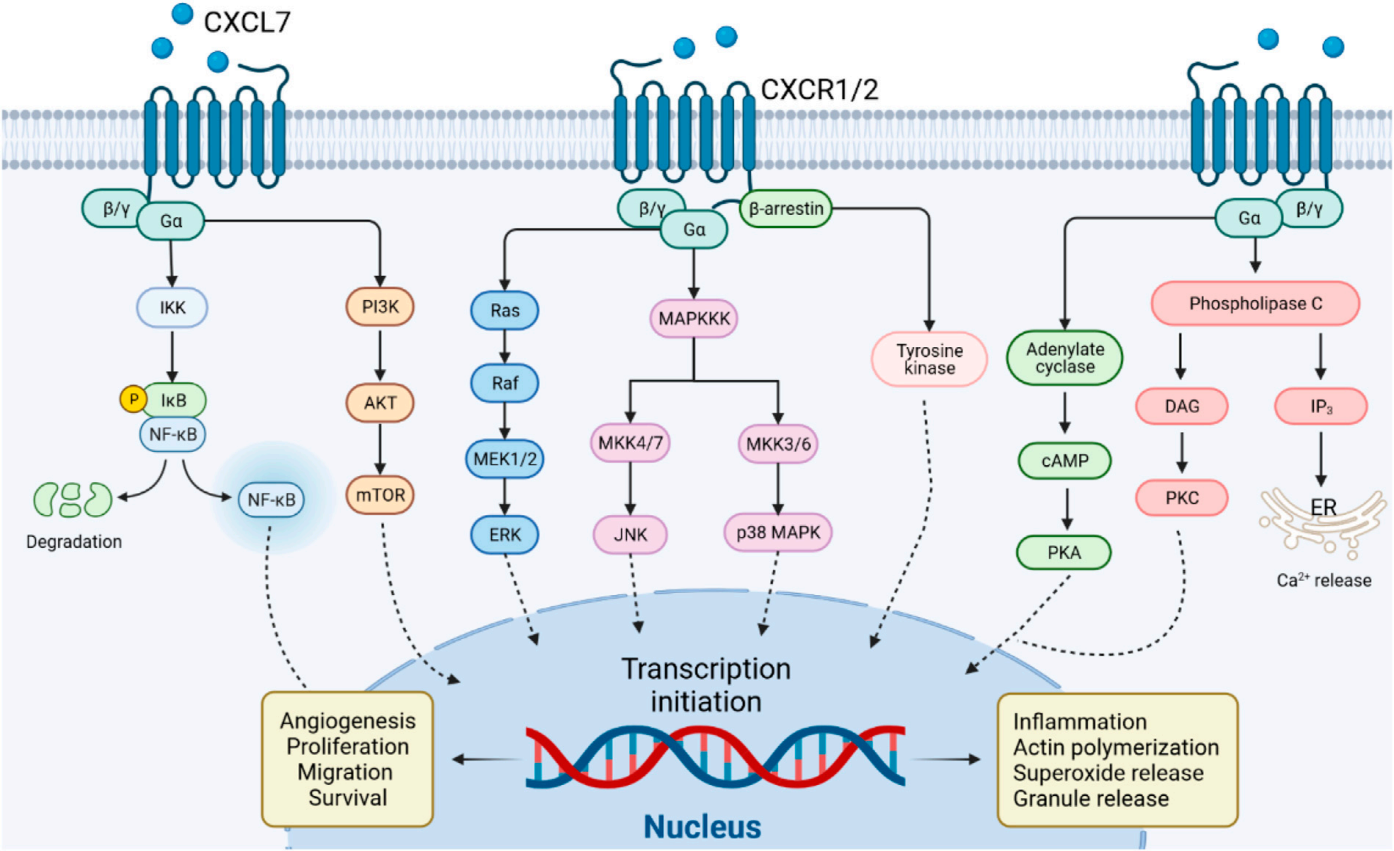
CXCL7/CXCR1/2 signal transduction pathway. CXCL7 binds to CXCR1/2 receptors via G protein or β-arrestin coupling to activate PI3K/AKT/mTOR, NF-κB, PKA, PKC or MAPK tertiary activation signaling pathways, mediating the role of CXCL7 in inflammation and tumor.

PI3K, the major downstream intracellular signal of CXCL7, induces phosphorylation of its substrate Akt. mTOR is a component of the AKT signaling pathway, which is a central regulator of cell metabolism, proliferation, growth, and survival (Alzahrani, 2019). mTOR is activated in various pathological cellular processes, such as tumor formation, angiogenesis, insulin resistance, and T lymphocyte activation (Wysocki, 2009). CXCL7 activates the PI3K/AKT/mTOR signaling pathway and promotes proliferation and migration of tumor cells (Grépin et al., 2014; Guo et al., 2017). CXCL7 induces tumor angiogenesis also through activation of the PI3K/AKT/mTOR pathway in endothelial cells (Figure 3) (Grépin et al., 2014).

### 5.2 The MAPK Signaling Pathway

The MAPK signaling cascade consists of multiple serine and threonine kinases. MAPK can be divided into four subfamilies, including ERK, p38 MAPK, JNK, and ERK5 (Fang and Richardson, 2005). The MAPK subfamily signaling pathways perform different duties: ERK regulates cell growth and differentiation, and the JNK and p38 MAPK signaling pathways exert important roles in stress responses such as apoptosis and inflammation (Santarpia et al., 2012; Arthur and Ley, 2013). CXCL7 activates the classical MAPK signaling pathway Ras/Raf/ERK, which promotes cell proliferation and angiogenesis in tumors (Grépin et al., 2014). CXCL7 also plays a role in inflammatory diseases through the JNK and p38 MAPK signaling pathways (Figure 3).

### 5.3 The NF-κB Signaling Pathway

The NF-κB signaling pathway may be stimulated by a variety of cytokines, growth factors, and tyrosine kinases (Hoesel and Schmid, 2013). Activated NF-κB signaling pathway regulates CXCL7 transcription and, in turn, CXCL7 influences inflammation and tumorigenesis *via* NF-κB (Figure 3) (Wang et al., 2021). In arthritis, CXCL7 induces NF-κB activation to promote the migration of fibroblastic synovial cells (Wang et al., 2021). This may be related to the NF-κB-induced expression of cell adhesion molecules (e.g. ICAM-1) and invasion-related proteins (Lawrence, 2009). Furthermore, activation of signaling pathways such as PI3K/Akt and Ras/MAPK can also regulate the function of the NF-κB pathway (Tang et al., 2022). The crosstalk of CXCL7 on the above signaling pathways needs to be further investigated.

### 5.4 The G Protein or β-arrestin Signaling Pathway

CXCL7 is a strong chemotactic agent of neutrophils, which activates G-protein or β-arrestin signaling pathways upon binding to CXCR1 or CXCR2, coordinating the recruitment of neutrophils to infected or injured tissues (Ludwig et al., 1997). Activation of G proteins induces the production of cyclic adenosine monophosphate (cAMP), inositol triphosphate (IP_3_) and diacylglycerol (DAG). β-arrestin induces MAPK and tyrosine kinase activation (Rajarathnam et al., 2019). The G protein and β-arrestin signaling cascades contribute to actin remodeling and integrin activation, mediating neutrophil adhesion to blood vessels and subsequent transendothelial migration (Detmers et al., 1991; Schenk et al., 2002). These pathway also induces the release of cytotoxic enzymes and ROS from neutrophil granules, which play an important role in the inflammatory response (Walz and Baggiolini, 1989). In general, G protein-mediated signaling starts rapidly and diminishes within minutes. In contrast, β-arrestin-mediated events are characterized by a slower onset and a longer duration (Luttrell and Gesty-Palmer, 2010). Due to selection bias, whether CXCL7 exerts its chemotactic effect on neutrophils through G-protein or β-arrestin cascade signaling after binding to CXCR1/CXCR2 needs to be further investigated (Figure 3).

### 6 Clinical Trials for CXCL7/CXCR1/2

Considering the role played by CXCL7/CXCR1/2 in inflammatory and neoplastic diseases, the development of drugs targeting the CXCL7/CXCR1/2 axis may benefit disease treatment. The corresponding drugs are now being tested in clinical trials with encouraging results (Table 2).

**TABLE 2:** Clinical trials for CXCL7/CXCR1/2.

Clinical Trial Code Number	Interventions	Condition	Title	Phase
NCT05254990	Reparixin	COVID-19 pneumonia Sars-CoV-2 infection	Reparixin as add-on Therapy to Standard of Care to Limit Disease Progression in Adult Patients With COVID-19	phase 3
NCT04794803	Reparixin	Severe pneumonia	Reparixin in COVID-19 Pneumonia - Efficacy and Safety	phase 2/3
NCT04878055	Reparixin	Pneumonia, Viral	Study on Efficacy and Safety of Reparixin in the Treatment of Hospitalized Patients With Severe COVID-19 Pneumonia	Phase 3
NCT05212701	Reparixin	Fatigue Locally advanced or metastatic breast Cancer	To Assess Efficacy and Safety of Oral Reparixin in Patients With Fatigue and Locally Advanced/Metastatic Breast Cancer	Phase 2
NCT01861054	Reparixin	Breast cancer	Pilot Study to Evaluate Safety and Biological Effects of Orally Administered Reparixin in Early Breast Cancer	Phase 2
NCT01220856	Reparixin	Pancreatic islet transplantation in type 1 diabetes mellitus	Reparixin in Pancreatic Islet Transplantation	Phase 2
NCT03031470	Reparixin	Ischemia-reperfusion injury in liver transplant Early allograft dysfunction	Pilot Study of Reparixin for Early Allograft Dysfunction Prevention in Liver Transplantation	Phase 2
NCT02001974	Reparixin	Metastatic breast cancer	Pilot Study to Evaluate Reparixin With Weekly Paclitaxel in Patients With HER 2 Negative Metastatic Breast Cancer (MBC)	Phase 1
NCT02370238	Reparixin	Metastatic breast cancer	A Double-blind Study of Paclitaxel in Combination With Reparixin or Placebo for Metastatic Triple-Negative Breast Cancer	Phase 2
NCT01967888	Reparixin	Pancreatectomy for chronic pancreatitis	Efficacy and Safety of Reparixin in Pancreatic Islet Auto-transplantation	Phase 2/3
NCT01817959	Reparixin	Islet transplantation in diabetes mellitus type 1	Study to Assess Efficacy and Safety of Reparixin in Pancreatic Islet Transplantation	Phase 3
NCT00248040	Reparixin	Ischemia-reperfusion injury Kidney diseases	Reparixin in Prevention of Delayed Graft Function After Kidney Transplantation	Phase 2
NCT00224406	Reparixin	Ischemia-reperfusion injury Lung transplantation	Repertaxin in Prevention of Primary Graft Dysfunction After Lung Transplantation	Phase 2
NCT03177187	AZD5069	Metastatic castration resistant prostate cancer	Combination Study of AZD5069 and Enzalutamide	Phase 1/2
NCT01480739	AZD5069	Chemokine receptor 2 (CXCR2) antagonist	AZD5069 Neutrophil Function Study	Phase 1
NCT01255592	AZD5069	Bronchiectasis Lung disease Respiratory diseases	Evaluation of the Effect of AZD5069 in Patients With Bronchiectasis	Phase 2
NCT01704495	AZD5069	Asthma	A Phase II Study to Evaluate the Efficacy, Safety and Tolerability of AZD5069 in Patients With Uncontrolled Persistent Asthma	Phase 2
NCT01233232	AZD5069	COPD Chronic bronchitis and emphysema	A 4 Week Study to Investigate the Safety and Tolerability of AZD5069 in Patients With Moderate to Severe Chronic Obstructive Pulmonary Disease (COPD)	Phase 2
NCT02583477	AZD5069	Metastatic pancreatic ductal adenocarcinoma	Phase Ib/II Study of MEDI4736 Evaluated in Different Combinations in Metastatic Pancreatic Ductal Carcinoma	Phase 1/2
NCT02499328	AZD5069	Advanced solid tumors Metastatic squamous cell carcinoma of the head and neck	Study to Assess MEDI4736 With Either AZD9150 or AZD5069 in Advanced Solid Tumors and Relapsed Metastatic Squamous Cell Carcinoma of Head and Neck	Phase 1/2
NCT00688467	SCH 527123	Asthma	Efficacy and Safety of Navarixin (SCH 527123) in Participants With Allergen-Induced Asthma (P05363)	Phase 2
NCT01068145	SCH 527123	Chronic obstructive pulmonary disease	Two-Part Study to Evaluate the Dose Response of SCH 527123 on Sputum Neutrophilia Following Ozone Challenge in Healthy Subjects and Chronic Obstructive Pulmonary Disease (COPD) Patients (P05567 a.m.7)	Phase 1
NCT01006616	SCH 527123	COPD	Long-Term Study of the Effects of Navarixin (SCH 527123, MK-7123) in Participants With Moderate to Severe COPD (MK-7123-019)	Phase 2
NCT03473925	SCH 527123	Solid tumors Non-small cell lung cancer Castration resistant prostate cancer Microsatellite stable colorectal cancer	Efficacy and Safety Study of Navarixin (MK-7123) in Combination With Pembrolizumab (MK-3475) in Adults With Selected Advanced/Metastatic Solid Tumors (MK-7123-034)	Phase 2
NCT01453478	GSK1325756	Pulmonary disease, chronic obstructive	A Study to Look at How GSK1325756 is Taken up by the Body When Given by Mouth When Stomach Acid is Reduced	Phase 1
NCT02169583	GSK1325756	Infections, respiratory tract	Study to Evaluate the Safety, Tolerability, and Pharmacokinetics of Single and Repeat Escalating Doses of GSK1325756 Solution for Infusion, and Absolute Bioavailability Relative of an Oral Dose, in Healthy Adult Subjects	Phase 1
NCT03250689	GSK1325756	Pulmonary disease, Chronic obstructive	Randomized Study Evaluating the Effect of Danirixin on Neutrophil Extracellular Traps (NETs) in Chronic Obstructive Pulmonary Disease (COPD)	Phase 2
NCT02469298	GSK1325756	Virus diseases	Safety, Tolerability and Clinical Effect of Danirixin in Adults With Influenza	Phase 2
NCT00748410	SB-656933	Colitis, Ulcerative	Study to Evaluate the Pharmacodynamics of SB-656933 in Patients With Ulcerative Colitis	Phase 2
NCT00615576	SB-656933	Pulmonary disease, Chronic obstructive	Repeat Dose Study in Male Healthy Volunteer Smokers	Phase 1
NCT01571895	DF2156A	Bullous pemphigoid	Pilot Efficacy and Safety Study of Oral DF2156A in Patients With Active Bullous Pemphigoid	Phase 2
NCT04245397	SX-682	Myelodysplastic syndromes	SX-682 Treatment in Subjects With Myelodysplastic Syndrome Who Had Disease Progression or Are Intolerant to Prior Therapy	Phase 1
NCT03161431	SX-682	Melanoma stage III Melanoma stage IV	SX-682 Treatment in Subjects With Metastatic Melanoma Concurrently Treated With Pembrolizumab	Phase 1
NCT04599140	SX-682	Metastatic colon adenocarcinoma Metastatic rectal Adenocarcinoma	SX-682 and Nivolumab for the Treatment of RAS-Mutated, MSS Unresectable or Metastatic Colorectal Cancer, the STOPTRAFFIC-1 Trial	Phase 1/2
NCT04477343	SX-682	Pancreatic cancer	A Study to Evaluate the Safety and Tolerability of SX-682 in Combination With Nivolumab as a Maintenance Therapy in Patients With Metastatic Pancreatic Ductal Adenocarcinoma	Phase 1
NCT04574583	SX-682	Metastatic cancer Solid tumors	Phase I/II Trial Investigating the Safety, Tolerability, Pharmacokinetics, Immune and Clinical Activity of SX-682 in Combination With BinTrafusp Alfa (M7824 or TGF-beta “Trap"/PD-L1) With CV301 TRICOM in Advanced Solid Tumors (STAT)	Phase 1/2

Reparixin is a noncompetitive metastable inhibitor of CXCR1 and CXCR2 with a 400-fold greater potency to inhibit CXCR1 activity than CXCR2 (Bertini et al., 2004). Clinical studies on reparixin have been completed evaluating its use for the treatment of ischemia-reperfusion injury, organ transplant-associated disease. The results showed that reparixin can effectively regulate neutrophil recruitment and attenuate inflammatory injury in ischemia-reperfusion (Opfermann et al., 2015). In the treatment of breast cancer, Reparixin has been shown to be safe and well-tolerated, and has shown benefit in reducing breast cancer tumor stem cells (Goldstein et al., 2020). Given the potent anti-inflammatory effects of reparixin, a number of clinical trials on the safety and efficacy of reparixin in the treatment of COVID-19 pneumonia are ongoing, as reparixin will hopefully be used as an adjuvant treatment for COVID-19 pneumonia.

AZD5069 is a selective CXCR2 antagonist. The clinical safety, tolerability, and efficacy of AZD5069 have been studied in acute and chronic inflammatory lung diseases and in advanced metastatic cancers (prostate and head and neck cancer). In inflammatory lung disease, AZD5069 treatment has been shown to be well tolerated in clinical trials with a reduction in neutrophil infiltration in blood and sputum. However, it was not associated with the improved clinical outcomes explored in the trial, so additional clinical trials are needed to explore its effectiveness (O'Byrne et al., 2016; De Soyza et al., 2015). AZD5069 has been shown to improve TGF-β signaling pathway-mediated adriamycin resistance through inhibition of CXCR2 in triple-negative breast cancer cells and to enhance atezolizumab immunotherapy efficacy (Ghallab et al., 2022). Therefore, the results of the ongoing clinical trials of AZD5069 antitumor therapy are worthy of our expectation.

Sch527123, also known as Navarixin, is a CXCR1 and CXCR2 antagonist with a high affinity for CXCR2. Current clinical studies on SCH527123 have focused on inflammatory diseases such as COPD, asthma and solid tumors (non-small cell lung cancer, prostate cancer and colon cancer). These studies evaluated the safety, efficacy and dose range of SCH527123 in the treatment of respective diseases. In a randomized, double-blind, placebo-controlled study, SCH527123 resulted in a significant reduction in ozone-induced airway neutrophils in healthy subjects, which was safe and well tolerated (Holz et al., 2010). However, further evaluation in a large trial in patients with pulmonary dysfunction is warranted.

GSK1325756 is a potent competitive CXCR2 receptor antagonist. Clinical trials evaluated the safety, tolerability, pharmacokinetics, and bioavailability studies of GSK1325756 in COPD and respiratory viral infectious diseases (Lazaar et al., 2018).

SB-656933 is a selective CXCR2 antagonist. Clinical studies have been conducted in airway inflammatory and intestinal inflammatory diseases. SB-656933 was shown to have a dose-dependent effect on neutrophil activation and recruitment in the tolerated dose range, making this drug potentially effective in neutrophil-dominant diseases (Lazaar et al., 2011; Moss et al., 2013).

SX-682 is a small molecule inhibitor of CXCR1 and CXCR2. Clinical trials of SX-682 are currently underway for the treatment of solid tumors (e.g., pancreatic and colon cancer) and non-solid tumors (e.g., myelodysplastic syndromes). SX-682 has been shown to inhibit MDSCs transport and enhance the immune efficacy of NK cells in head and neck tumor models (Greene et al., 2020).

## 7 Conclusion

Aberrant expression of CXCL7 in inflammatory diseases and tumors may serve as a biomarker for disease diagnosis and prognosis. CXCL7 acts as an inflammatory cytokine that induces various immune cells to participate in the pathogenesis of inflammatory diseases. In various tumors, CXCL7 has been preliminarily found to affect tumor growth, invasion, metastasis and tumor angiogenesis to some extent. CXCL7 binding to receptors CXCR1 or CXCR2 mediates its role in inflammatory diseases and tumors through signaling pathways such as PI3K/AKT/mTOR, NF-κB or MAPK. Currently, clinical trials have demonstrated the efficacy and safety of targeted CXCL7/CXCR1/CXCR2 therapy, which is encouraging for future clinical applications.

## Glossary

CXCL7: C-X-C motif ligand 7
CXCR1: C-X-C chemokine receptor 1
CXCR2: C-X-C chemokine receptor 2
NAP2: neutrophil-activating peptide 2
PPBP: pro-platelet basic protein
CTAP-III: connective tissue-activating peptide III
βTG-Ag: β-thromboglobulin antigen
GAG: glycosaminoglycan
VEGF: vascular endothelial growth factor
ALI/ARDS: acute lung injury/acute respiratory distress syndrome
BALF: bronchoalveolar lavage fluid
COVID-19: new coronavirus pneumonia
AUC: area under the curve
BPD: Bronchopulmonary dysplasia
UCB: umbilical cord blood
SIRS: systemic inflammatory response syndrome
ROS: reactive oxygen species
NOX2: NADPH oxidase 2
NET: extracellular neutrophil traps
OLA: open lung approach
PEEP: positive end-expiratory pressure
RM: recruitment maneuver
COPD: Chronic obstructive lung disease
ICAM-1: intercellular adhesion molecule 1
ELAM-1: endothelial leukocyte adhesion molecule 1
IKK: I-kappa B kinase
HASM: human airway smooth muscle
DCV: delayed cerebral vasospasm
CSF: cerebrospinal fluid
MSCs: mesenchymal stem cell
PKCδ: protein kinase C-delta
STAT3: signal transducer and activator of transcription 3
IBD: inflammatory bowel disease
CD: Crohn’s disease
UC: ulcerative colitis
MMP3: matrix metalloproteinase 3
RA: rheumatoid arthritis
STING: stimulator-of-interferon-gene
CHIKV: chikungunya virus
PEVs: platelet-derived extracellular vesicles
RA-FLS: rheumatoid arthritis fibroblast-like synoviocytes
GVHD: graft-versus-host disease
HSCT: hematopoietic stem cell transplantation
NMO: optic neuromyelitis optica
MS: multiple sclerosis
IIDDs: idiopathic inflammatory demyelinating diseases
ADEM: acute disseminated encephalomyelitis
uPA: urokinase plasminogen activator
LTD_4_: leukotrienes D_4_
LTC_4_: leukotrienes C_4_
CysLT_2_R: cysteinyl leukotriene receptor 2
ERK: extracellular signal-regulated kinase
JNK: c-Jun terminal kinase
p38 MAPK: p38 mitogen-activated protein kinase
MAPK: mitogen-activated protein kinase
APS: antiphospholipid syndrome
VKH: Vogt-Koyanagi-Harada syndrome
RAEB: refractory anemia with excess blasts
RAEB-t: refractory anemia with excess blasts in transformation
ALL: acute lymphoblastic leukemia
AML: acute myeloid leukemia
LSCC: lung squamous cell carcinoma
NSCL: non-small cell lung cancer
ccRCC: clear cell renal cell carcinoma
RCC: renal cell carcinoma
IL-1β: interleukin 1β
CA Ⅸ: carbonic anhydrase 9
Gals-3: galectin-3
MMP9: matrix metalloproteinase 9
CC: cholangiocarcinoma
OS: overall survival
ICC: intrahepatic cholangiocarcinoma
TME: tumor microenvironment
CSCs: cancer stem cells
PFPE: piperine fractional piper nigrum extract
CRC: colorectal cancer
OCRC: obstructive colorectal cancer
DFS: disease-free survival
MDSCs: myeloid-derived suppressor cells
TAMs: tumor-associated macrophages
AATs: anti-angiogenic therapies
FGL2: fibrinogen-like protein 2
MEIS1: myeloid ecotropic viral integration site 1
BLCA: bladder cancer
LLC: Lewis lung cancer
HIF-1: hypoxia-inducible factors 1
cAMP: cyclic adenosine monophosphate
IP_3_: inositol triphosphate
DAG: diacylglycerol
